# Identification of Six Autophagy-Related-lncRNA Prognostic Biomarkers in Uveal Melanoma

**DOI:** 10.1155/2021/2401617

**Published:** 2021-08-12

**Authors:** Yao Chen, Lu Chen, Jinwei Wang, Jia Tan, Sha Wang

**Affiliations:** Hunan Key Laboratory of Ophthalmology, Eye Center of Xiangya Hospital, Central South University, China

## Abstract

Currently, no autophagy-related long noncoding RNA (lncRNA) has been reported to predict the prognosis of uveal melanoma patients. Our study screened for autophagy-related lncRNAs in 80 samples downloaded from The Cancer Genome Atlas (TCGA) database through lncRNA-mRNA coexpression. We used univariate Cox to further filter the lncRNAs. Multivariate Cox regression and LASSO regression were applied to construct an autophagy-associated lncRNA predictive model and calculate the risk score. Clinical risk factors were validated using Cox regression to determine whether they were independent prognostic indicators. Functional enrichment was performed using Gene Ontology and Kyoto Encyclopedia of Genes and Genomes. The model was built with six predictive autophagy-associated lncRNAs and clustered uveal melanoma patients into high- and low-risk groups. The risk score of our model was a significant independent prognostic factor (hazard ratio = 1.0; *p* < 0.001). Moreover, these six lncRNAs were significantly concentrated in the biological pathways of cytoplasmic component recycling, energy metabolism, and apoptosis. Thus, the six autophagy-associated lncRNAs are potential molecular biomarkers and treatment targets for uveal melanoma patients.

## 1. Introduction

Uveal melanoma is the most common intraocular malignancy in adults; the 10-year mortality rate of uveal melanoma is approximately 40%. Metastasis occurs in almost half of uveal melanoma patients, primarily in the liver. The survival time decreases to less than 1 year once metastasis is discovered [1]. Researchers have identified biomolecular abnormalities associated with a poor prognosis in uveal melanoma, such as the presence of monosomy 3 and gain of chromosome 8 [2]. Gene expression profiling divided the cancer into Class 1 and Class 2 based on the risk of metastasis [3] and revealed metastasis-related genetic mutations in BAP1, GNAQ/GNA11, EIF1AX, and SF3B1 [4, 5]. Despite the progress in understanding genetic regulation in uveal melanoma, current treatment modalities for this disease, such as brachytherapy, charged-particle radiotherapy, proton beam therapy, photodynamic therapy, and surgical excision, are still not beneficial to overall survival [6]. New strategies and potential targets are imperative to treat uveal melanoma.

Autophagy is a catabolic process involving the multistep degradation of proteins and organelles; it participates in maintaining cellular homeostasis, which is associated with heart disease, senescence, neurodegeneration, and cancer development [7]. Current studies of autophagy in uveal melanoma, though few, expose a new frontier to understand its carcinogenic and metastatic molecular mechanisms. Upregulated autophagy in uveal melanoma through intensified hypoxia is associated with metastasis and a poor prognosis [8]. However, enhanced autophagy in other studies inhibits cell proliferation and tumor growth [9]. Recently, Li et al. found that lncRNA ZNMT1 inhibited the tumorigenesis of uveal melanoma by inducing autophagy [10]. However, no other autophagy-associated lncRNA was reported [10].

Long noncoding RNAs (lncRNAs) are RNAs with a transcript length longer than 200 nucleotides that do not encode proteins; they play pivotal roles in epigenetic modification, chromatin remodeling, and genetic imprinting [11]. Gene sequencing of cancer has identified several protein-encoding genes as potential antitumor targets, with 98% of the sequence in noncoding regions, which indicates that most mechanisms of lncRNAs remain unclarified in cancer research [12]. Current studies have unraveled a close relationship between lncRNAs and uveal melanoma [13–15]. lncRNA interacts with microRNA (miRNA) and promotes uveal melanoma cell proliferation, tumor initiation, and metastasis by targeting EZH2 or through the p53 signaling pathway [13]. lncRNA such as SNHG15 indicates a poor prognosis in uveal melanoma, while TCONS_00004101, RP11-551L14.4, and TCONS_00004845 are metastasis-associated lncRNAs of uveal melanoma [16]. Autophagy-related lncRNAs have been studied extensively in several biological pathways and represent a new frontier for cancer study [17]. However, autophagy-associated lncRNAs in uveal melanoma have been rarely studied. This study was aimed at building an autophagy-associated lncRNA profile using TCGA database, investigating new lncRNA predictive biomarkers, and identifying potential molecular targets for uveal melanoma.

## 2. Materials and Methods

### 2.1. Data Extraction

The transcriptome RNA sequencing and clinical data of 80 uveal melanoma samples were downloaded from The Cancer Genome Atlas (TCGA) data portal (https://portal.gdc.cancer.gov/). The study excluded samples with a follow-up time of less than 30 days because these patients might have died of unpredictable factors. The raw data were collected, standardized by log2 transformation, and merged into a matrix file. The lncRNA profiling data was acquired from the RNA-seq dataset using Perl language. The Ensembl ID numbers of the genes were transformed to gene symbols using Perl language based on the Ensembl database.

### 2.2. Identification of Autophagy-Related Genes and lncRNA/mRNA Coexpression Network

The autophagy-related genes were downloaded from the Human Autophagy Database (HADb, http://www.autophagy.lu/) and gene set enrichment analysis (GSEA, https://www.gsea-msigdb.org/gsea/index.jsp). Pearson's correlation analysis was conducted using R software 3.6.2 to calculate the correlation of lncRNAs and autophagy-related genes. lncRNAs with squared correlation coefficient *R*^2^ > 0.3 and *p* < 0.001 were considered correlated with autophagy. Visualization of lncRNA/mRNA coexpression network was performed by Cytoscape software 3.6.1.

### 2.3. Establishment of Autophagy-Related lncRNA Biomarkers

Univariate Cox regression was applied to identify the prognostic value of autophagy-related lncRNAs, in which *p* value < 0.05 was incorporated into the least absolute shrinkage and selection operator (LASSO) regression [18] with the glmnet R package. Multivariate Cox regression analysis based on the results of LASSO regression was performed to establish a risk score and identify the prognostic lncRNA biomarkers. The risk score was established from the expression levels multiplied by the Cox regression coefficients: risk score = (0.34184∗SOS1 − IT1) + (1.14771∗AC016747.1) + (0.55510∗AC100791.3) − (2.78048∗AC104825.1) − (1.59941∗AC090617.5) + (0.33215∗AC018904.1). Patients with survival data were divided into high-risk and low-risk groups according to the median risk score. Kaplan–Meier survival analysis was applied to evaluate the predictive ability of the autophagy-related lncRNA biomarkers. Moreover, the relationship between the prognostic biomarkers and clinical features such as gender, age, tumor stage, and T stage from the TNM staging method (a method to describe the tumor status) was evaluated using univariate and multivariate Cox proportional hazard regression analyses.

### 2.4. Construction of a Predictive Nomogram [19]

We constructed a nomogram to predict the survival of uveal melanoma patients (1-, 3-, and 5-year survival). The concordance index (*C*-index), calibration plot, and receiver operating characteristic (ROC) analysis were used to validate the biomarkers.

### 2.5. Gene Ontology (GO) and Kyoto Encyclopedia of Genes and Genomes (KEGG) Enrichment Analysis

We used GSEA v3.0 software [20, 21] to identify the top 10 KEGG signaling pathways and functionally enriched GO terms regulated by the autophagy-related biomarkers.

### 2.6. Cell Culture

Normal human uveal melanocytes (UM-U-95) were donated while uveal melanoma cell line MP46 (CRL-3298) was purchased from ATCC. The cells were cultured in DMEM with 10% heat-inactivated fetal bovine serum at 37°C incubator and 5% CO_2_ in air atmosphere.

### 2.7. qRT-PCR

We utilized the qRT-PCR to validate the expression of these lncRNA biomarkers between normal human uveal melanocytes and uveal melanoma. cDNA of each sample was converted for qPCR. The primers were designed by Primer3 targeting each lncRNA biomarker. U6 was utilized for the housekeeping gene. The cycling condition was conducted as follows: 94°C for denaturation, 60°C for annealing, and 72°C for extension. All PCR was performed in LightCycler 480 (Roche). Primers are listed in supplementary table 2.

### 2.8. Statistical Analysis

Differences between groups were compared using R software (Wilcoxon test). LASSO regression analysis and univariate and multivariate Cox regression analyses were applied to identify prognostic lncRNA biomarkers for uveal melanoma patients. Kaplan–Meier analysis was used to build survival curves, and the significance of the differences in survival time was calculated using the log-rank test. *p* < 0.05 was considered statistically significant.

## 3. Results

### 3.1. Screening for Autophagy-Associated lncRNAs in Uveal Melanoma Patients

We identified 14142 lncRNAs from the RNA sequencing data of TCGA-UVM. In total, 516 autophagy-associated genes were obtained from GSEA and the HADb database (Supplementary table 1). We constructed a lncRNA-mRNA coexpression network to screen for autophagy-associated lncRNAs. 730 lncRNAs were selected through Pearson's correlation analysis with the criteria of ∣*R*^2^ | >0.3 and *p* < 0.001.

### 3.2. Coexpression of lncRNA/mRNA and Construction of the Autophagy-Associated lncRNA Model in Uveal Melanoma

**Figure 1:**
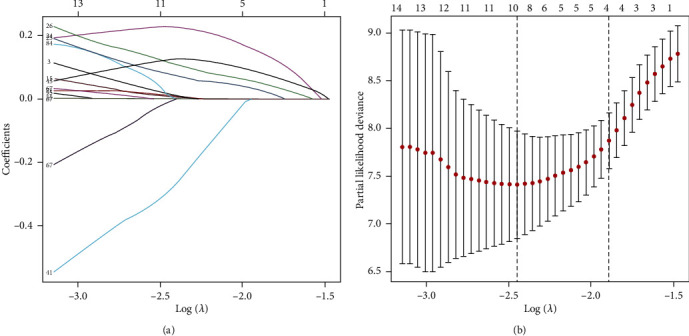
LASSO model. Autophagy-related lncRNA selection using the LASSO model. (a) Shows the profiles of LASSO coefficients. (b) Shows the LASSO coefficient values of the 6 autophagy-related lncRNAs in uveal melanoma. The vertical dashed lines are the optimal log(*λ*) values.

**Figure 2:**
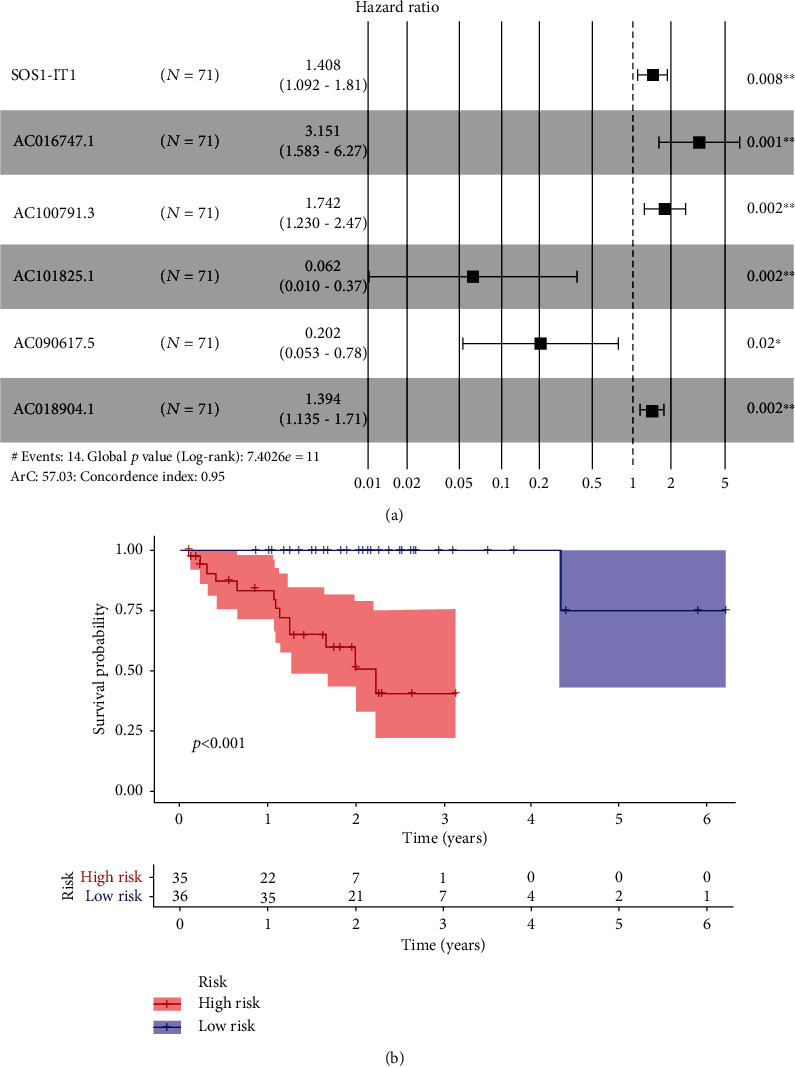
The selected biomarkers and prognosis: (a) forest map of the relationships among the six autophagy-associated lncRNAs and prognosis in uveal melanoma patients; (b) overall survival of the six-lncRNA signatures in uveal melanoma patients.

We identified 105 autophagy-associated lncRNAs with a prognostic value in uveal melanoma patients based on the results of Cox univariate analysis (*p* < 0.05) and then selected six prognosis-related lncRNAs by LASSO regression analysis (Figure 1). A coexpression network of lncRNAs and mRNAs was constructed (Supplementary Fig. 2B). As shown in the Sankey diagram (Supplementary Fig. 2A), among these lncRNAs, SOS1-IT1, AC016747.1, AC100791.3, and AC018904.1 are risky prognostic lncRNAs, whereas AC104825.1 and AC090617.5 are protective prognostic lncRNAs. The risk score was calculated with the following formula: risk score = (0.34184∗SOS1 − IT1) + (1.14771∗AC016747.1) + (0.55510∗AC100791.3) − (2.78048∗AC104825.1) − (1.59941∗AC090617.5) + (0.33215∗AC018904.1). The relationship between the selected lncRNAs and prognosis is shown in the forest map (Figure 2(a)).

### 3.3. Evaluation of the Prognostic Biomarkers

**Figure 3:**
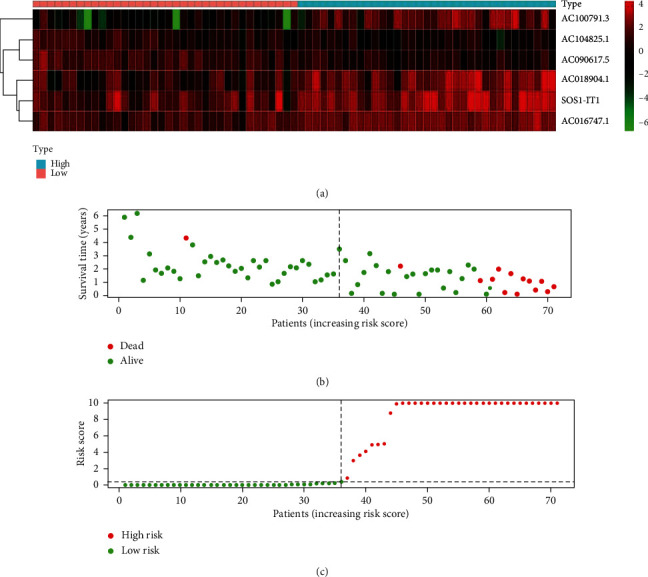
Risk score: (a) heatmap of the expression of the six lncRNAs in uveal melanoma patients; (b) distribution of the survival time and status in relation to risk score; (c) distribution of high- and low-risk patients according to the risk score.

**Figure 4:**
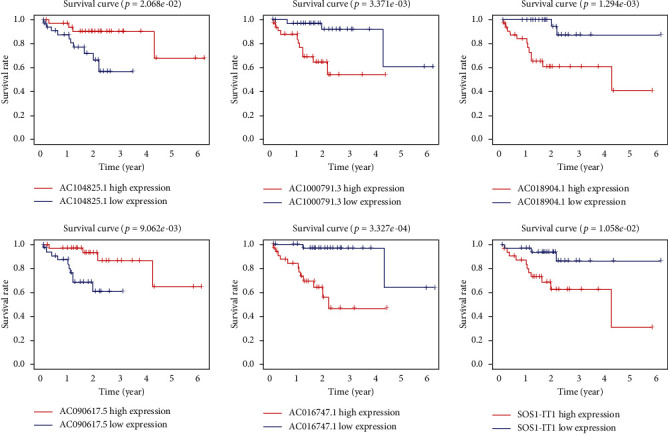
The association between the selected lncRNAs and survival. Expression of the selected six lncRNAs and their relationship to the survival rate of uveal melanoma patients.

To evaluate the aforementioned autophagy-lncRNA prognostic model, we divided the uveal melanoma patients into high- and low-risk groups according to the median risk score of the selected six autophagy-related lncRNAs. The number of patient deaths increased with increasing risk score (Figure 3(b)). The overall survival was longer in the low-risk group than in the high-risk group (*p* < 0.001) (Figure 3(c)). Regarding individual lncRNAs of the prognostic signature, the survival rate was also significantly associated with the expression of each lncRNA (patients were divided into high- and low-expression groups based on the median expression of lncRNA) (Figure 4).

### 3.4. Prognostic Associations of the Selected lncRNA Biomarkers for Clinicopathological Features

**Figure 5:**
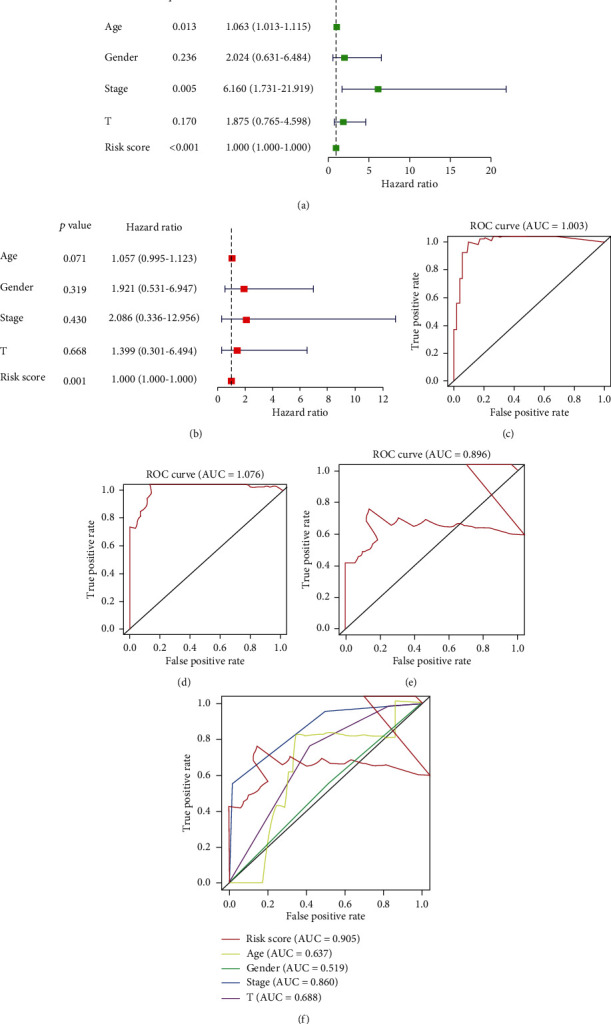
Risk factors. Univariate (a) and multivariate (b) Cox regression analyses of clinical features, such as age, gender, stage, T stage, and risk score. The 1-year (c), 3-year (d), and 5-year (e) ROC curves of the risk scores and ROC curves of the clinical features (f).

**Figure 6:**
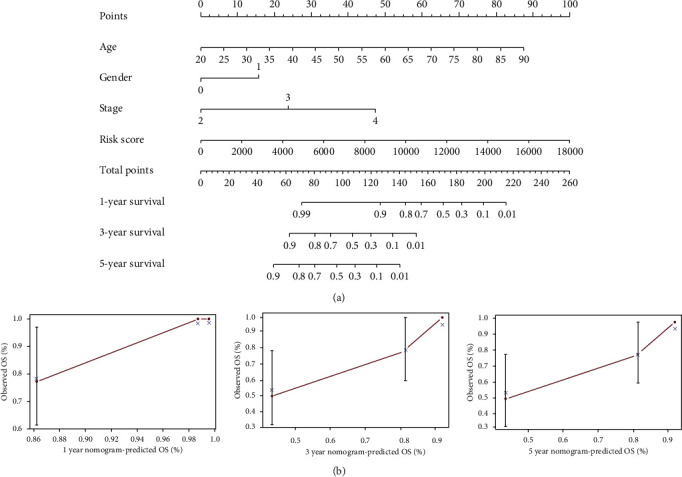
Overall survival. Nomogram for the overall survival prediction at 1, 3, and 5 years (a) and calibration plots of the nomogram at 1, 3, and 5 years (b).

We studied the relationship between the risk score of autophagy-associated lncRNA biomarkers and clinicopathological characteristics of the uveal melanoma patients, such as age, gender, and tumor stage. The risk score was significantly increased if the patient was older than 60 years. Univariate Cox regression was performed to identify three independent prognostic indicators (age, stage, and risk score) (Figure 5(a)). Multivariate Cox regression revealed that risk score was a strong independent prognostic factor for uveal melanoma survival (Figure 5(b)). The subsequent calculated AUCs for risk score, age, gender, stage, and stage T of the ROC curves were 0.905, 0.637, 0.519, 0.860, and 0.688, respectively, which demonstrates that the risk score and stage were two influential indicators (Figure 5(f)). The nomogram for overall survival prediction at 1, 3, and 5 years was constructed by integrating these clinicopathological features, and calibration plots of the nomogram suggested consistency between observation and prediction (Figure 6). The *C*-index was 0.912 for the nomogram. ROC analysis showed that the AUCs of the nomogram at 1-, 3-, and 5-year survival were 1.003, 1.076, and 0.896, respectively, which indicates a favourable predictive capability of our model.

### 3.5. Gene Set Enrichment Analysis of the lncRNA Biomarkers

**Figure 7:**
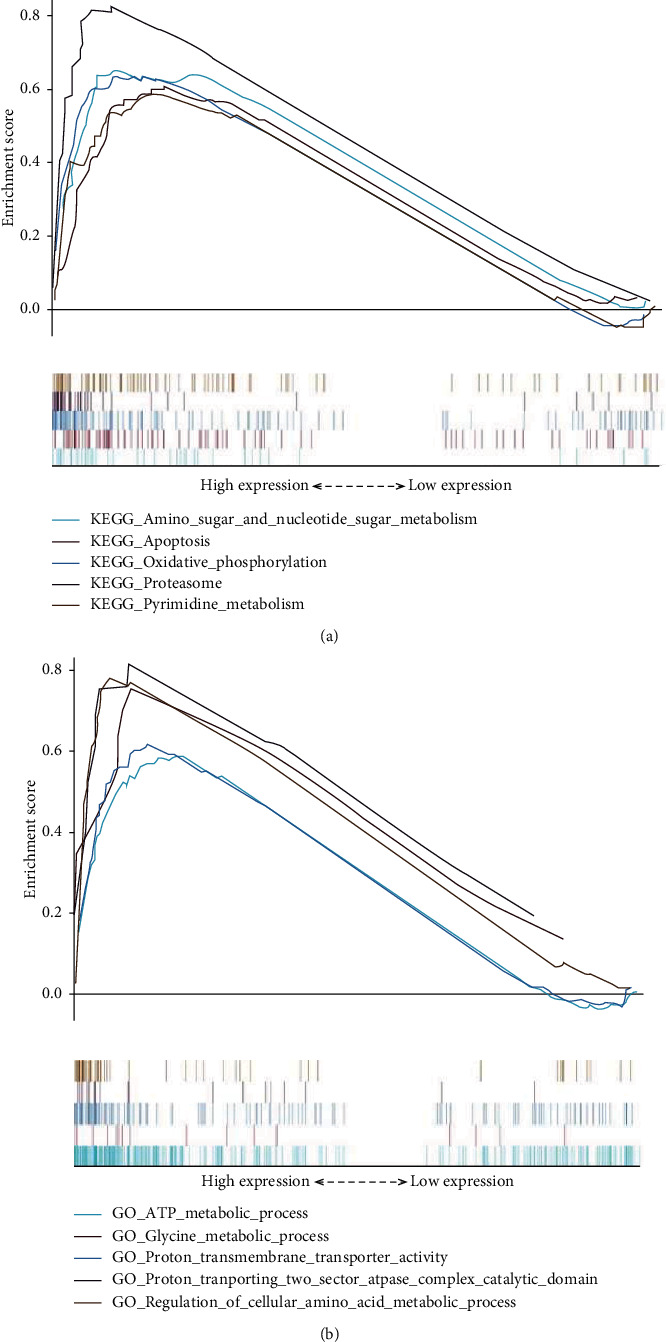
Gene set enrichment analysis of the lncRNA biomarkers. The top five enriched gene sets are shown from gene set enrichment analysis of the autophagy-associated lncRNA biomarkers performed using gene sets of GO (a) and KEGG (b).

**Figure 8:**
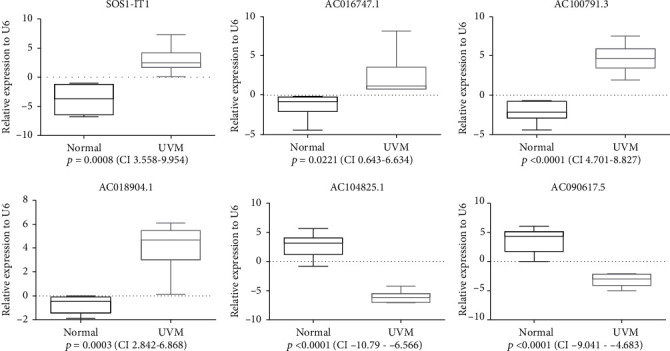
RT-PCR results. The expression of six lncRNAs in uveal melanoma and normal uveal melanocyte. Normal; human uveal melanocytes; UVM: human uveal melanoma cells: *P*: *p* value; CI: confidence interval.

Gene set enrichment analysis of the autophagy-associated lncRNA biomarkers was performed using gene sets in GO and KEGG. GO analysis showed that the autophagy-associated lncRNAs were mostly enriched in cellular ATP metabolic process, protein metabolic process, and proton transporting activity (Figure 7(a)). KEGG analysis indicated that the autophagy-associated lncRNAs were concentrated in molecular signaling pathways such as amino sugar and nucleotide sugar metabolism, proteasome, apoptosis, and oxidative phosphorylation (Figure 7(b)). To further verify the six lncRNA biomarkers, we tested these six autophagy-associated lncRNAs' expression between normal uveal melanocyte and uveal melanoma. Our qRT-PCR results demonstrated that the expression of SOS1-IT1, AC016747.1, AC100791.3, and AC018904.1 was higher in uveal melanoma cell line; in contrast, uveal melanoma cell lines had a significantly lower expression of AC104825.1 and AC090617.5 (Figure 8) with *p* value < 0.05. These results confirmed the clinical utility of six autophagy-associated lncRNAs as biomarkers for uveal melanoma.

## 4. Discussion

Uveal melanoma is the most common primary intraocular tumor in adults, and progress has been made in genetic prognostic testing, with gene expression profiling clustering the tumor into Class 1 (high metastatic potential) and Class 2 (low metastatic potential) tumors [3]. Additionally, researchers have reported miRNA signatures to predict uveal melanoma prognosis based on bioinformatics analysis [22, 23]. However, studies using real-world samples of uveal melanoma reported no association between the miRNAs and overall survival or metastasis [24]. Therefore, more effective biomarkers must be investigated.

Autophagy is a highly conversed cellular process that maintains the energy level to recycle amino acid and other nutrients, as well as renew cytoplasmic constituents [25]. Autophagy has dual functions in tumorigenesis: in normal cells, autophagy plays a pivotal role in surveilling damaged organelles, purging congregated proteins, and reducing abnormal DNA and reactive oxygen compounds, preventing somatic cells from transforming into cancer cells [26–28]. Conversely, in tumor cells, the aforementioned functions of autophagy inversely accelerate the metabolism rate, enhance the cellular capability of taking up nutrients, resist apoptosis, and develop multidrug resistance, which subsequently propels cancer development [29, 30]. In uveal melanoma patients, autophagy-associated proteins MAP1LC3A and BECN1 are commonly upregulated and are related to tumor development which resulted in poor prognosis [8]. In uveal melanoma cell lines, increased autophagy helps tumor cell survive stressed conditions [9], which indicates the vital role of autophagy in uveal melanoma progression.

In recent years, several bioinformatics studies have been performed on uveal melanoma with the development of high-throughput sequencing to identify useful indicators for prognosis or therapeutic targets, including genome-wide predictors, methylation biomarkers, and prognostic lncRNAs [31–33]. In particular, since the discovery of lncRNAs' function as important regulators in multiple cellular processes, lncRNAs have become a hotspot in cancer studies. However, autophagy-associated lncRNAs have been less reported in uveal melanoma and a detailed and thorough analysis of the associations between lncRNA expression and autophagy in uveal melanoma prognosis remains poorly understood.

We identified six autophagy-associated lncRNAs to predict the prognosis of uveal melanoma by screening lncRNAs from 80 tumor samples from TCGA database. Clinical features such as age older than 60 years and stage were independent risk factors. Furthermore, the functions of these lncRNAs are concentrated on cellular energy and nutrient metabolism and protease activity based on GO and KEGG analyses, consistent with the functions of autophagy.

Among these six lncRNAs, SOS1-IT1, AC016747.1, AC100791.3, and AC018904.1 are hazardous indicators, while AC104825.1 and AC090617.5 are protective factors. SOS1-IT1 was also reported as a risk factor in an ivermectin-related three-lncRNA prognostic model of ovarian cancer [34]. According to the description on GeneCards (https://www.genecards.org/), it is sense intronic to MAP4K3, which is a serine/threonine kinase leading to T-cell activation, cellular autophagy inhibition, cancer recurrence, and metastasis [35]. AC016747.1, also known as LOC339803, acts as a ceRNA of miR-30a-5p and promotes the migration and invasion of hepatocellular carcinoma [36]. Additionally, it is highly expressed in human atherosclerotic lesions to enhance transcription factors in abnormal endothelial metabolism when subjected to hypoxia [37]. AC100791.3 is antisense to TBC1D16 according to GeneCards, with the protein TBC1D16 playing a critical role in the progression of cutaneous melanoma [38]. AC018904.1, although its function is unclear, is described as antisense to ADAM10 on GeneCards. ADAM10, as a disintegrin and metalloproteinase 10, participates in multiple catalytic activities, including apoptosis, autoimmunity, cell adhesion and metabolism, cancer proliferation, and metastasis [39]. Increased ADAM10 impairs autophagy and aggravates inflammatory activities in fibroblasts. However, in Alzheimer's disease, ADAM10 facilitates autophagy in cleaving and removing abnormal proteins, demonstrating a neuroprotective effect [40]. These facts imply a potential effect of AC018904.1 on autophagy. No data have been reported for the remaining two lncRNAs AC104825.1 and AC090617.5 or their aliases on GeneCards. Further study is warranted on the above six lncRNAs to unravel the underlying mechanisms between autophagy and uveal melanoma, and they could also be used as possible biologic targets.

However, this study has some limitations. First, the 80 samples in TCGA database were relatively small compared with the patient number of other cancer types, leading to deviation in the analysis. Second, because most of the clinical data of the M and N stages of uveal melanoma patients are not available, whether they are independent risk factors is unclear. Third, the prognostic value of our model should be further validated and confirmed by other cohorts. Fourth, other prognostic features, such as ciliary body involvement, tumor height, epithelioid cell type, and chromosomal status, were not included in our study because some of the information was not available.

## 5. Conclusions

Our study filtered six autophagy-related lncRNAs through TCGA database. The risk model based on these six lncRNAs could cluster the prognosis of uveal melanoma into high- and low-risk groups. More importantly, the risk score could be used as an independent risk factor for the prediction of overall survival and provide evidence for potential biomarkers in uveal melanoma treatment.

## Data Availability

The data used during the study are available online (TCGA database, https://portal.gdc.cancer.gov/; HADb database, http://www.autophagy.lu/; and GSEA database, https://www.gsea-msigdb.org/gsea/index.jsp).

## Supplementary Materials

Supplementary 1 Supplementary Table 1: a total of 516 autophagy-associated genes obtained from GSEA and the HADb database.

Supplementary 2 Supplementary Table 2: primers for rtPCR.

Supplementary 3 Supplementary Figure: coexpression. Coexpression of lncRNA/mRNA shown with a Sankey diagram (A) and Cytoscape (B).
