# Supplementary material for: Identification of Six Autophagy-Related-lncRNA Prognostic Biomarkers in Uveal Melanoma
Source: Dis Markers. 2021 Aug 12;2021:2401617. doi: 10.1155/2021/2401617 (PMC8379639; doi:10.1155/2021/2401617)
Supplement: Supplementary 1 — Supplementary Table 1: a total of 516 autophagy-associated genes obtained from GSEA and the HADb database. [file 2401617.f1.docx]

Supplementary Table 1: A total of 516 autophagy-associated genes obtained from GSEA and the HADb database.

| Autophagy-associated genes |
| --- |
| ABL1 |
| ACBD5 |
| ACIN1 |
| ACTRT1 |
| ADAMTS7 |
| AKR1E2 |
| ALKBH5 |
| ALPK1 |
| AMBRA1 |
| ANXA5 |
| ANXA7 |
| ARSB |
| ASB2 |
| ATG10 |
| ATG12 |
| ATG13 |
| ATG14 |
| ATG16L1 |
| ATG16L2 |
| ATG2A |
| ATG2B |
| ATG3 |
| ATG4A |
| ATG4B |
| ATG4C |
| ATG4D |
| ATG5 |
| ATG7 |
| ATG9A |
| ATG9B |
| ATP13A2 |
| ATP1B1 |
| ATPAF1-AS1 |
| ATPIF1 |
| BECN1 |
| BECN1P1 |
| BLOC1S1 |
| BMP2KL |
| BNIP1 |
| BNIP3 |
| BOC |
| C11orf2 |
| C11orf41 |
| C12orf44 |
| C12orf5 |
| C14orf133 |
| C1orf210 |
| C5 |
| C6orf106 |
| C7orf59 |
| C7orf68 |
| C8orf59 |
| C9orf72 |
| CA7 |
| CALCB |
| CALCOCO2 |
| CAPS |
| CCDC36 |
| CD163L1 |
| CD93 |
| CDC37 |
| CDKN2A |
| CHAF1B |
| CHMP2A |
| CHMP2B |
| CHMP3 |
| CHMP4A |
| CHMP4B |
| CHMP4C |
| CHMP6 |
| CHST3 |
| CISD2 |
| CLDN7 |
| CLEC16A |
| CLN3 |
| CLVS1 |
| COX8A |
| CPA3 |
| CRNKL1 |
| CSPG5 |
| CTSA |
| CTSB |
| CTSD |
| CXCR7 |
| DAP |
| DKKL1 |
| DNAAF2 |
| DPF3 |
| DRAM1 |
| DRAM2 |
| DYNLL1 |
| DYNLL2 |
| DZANK1 |
| EI24 |
| EIF2S1 |
| EPG5 |
| EPM2A |
| FABP1 |
| FAM125A |
| FAM131B |
| FAM134B |
| FAM13B |
| FAM176A |
| FAM176B |
| FAM48A |
| FANCC |
| FANCF |
| FANCL |
| FBXO7 |
| FCGR3B |
| FGF14 |
| FGF7 |
| FGFBP1 |
| FIS1 |
| FNBP1L |
| FOXO1 |
| FUNDC1 |
| FUNDC2 |
| FXR2 |
| GABARAP |
| GABARAPL1 |
| GABARAPL2 |
| GABARAPL3 |
| GABRA5 |
| GDF5 |
| GMIP |
| HAP1 |
| HAPLN1 |
| HBXIP |
| HCAR1 |
| HDAC6 |
| HGS |
| HIST1H3A |
| HIST1H3B |
| HIST1H3C |
| HIST1H3D |
| HIST1H3E |
| HIST1H3F |
| HIST1H3G |
| HIST1H3H |
| HIST1H3I |
| HIST1H3J |
| HK2 |
| HMGB1 |
| HPR |
| HSF2BP |
| HSP90AA1 |
| HSPA8 |
| IFI16 |
| IPPK |
| IRGM |
| IST1 |
| ITGB4 |
| ITPKC |
| KCNK3 |
| KCNQ1 |
| KIAA0226 |
| KIAA1324 |
| KRCC1 |
| KRT15 |
| KRT73 |
| LAMP1 |
| LAMP2 |
| LAMTOR1 |
| LAMTOR2 |
| LAMTOR3 |
| LARP1B |
| LENG9 |
| LGALS8 |
| LIX1 |
| LIX1L |
| LMCD1 |
| LRRK2 |
| LRSAM1 |
| LSM4 |
| MAP1A |
| MAP1LC3A |
| MAP1LC3B |
| MAP1LC3B2 |
| MAP1LC3C |
| MAP1S |
| MAP2K1 |
| MAP3K12 |
| MARK2 |
| MBD5 |
| MDH1 |
| MEX3C |
| MFN1 |
| MFN2 |
| MLST8 |
| MRPS10 |
| MRPS2 |
| MSTN |
| MTERFD1 |
| MTMR14 |
| MTMR3 |
| MTOR |
| MTSS1 |
| MYH11 |
| MYLK |
| MYOM1 |
| NBR1 |
| NDUFB9 |
| NEFM |
| NHLRC1 |
| NME2 |
| NPC1 |
| NR2C2 |
| NRBF2 |
| NTHL1 |
| NUP93 |
| OBSCN |
| OPTN |
| P2RX5 |
| PACS2 |
| PARK2 |
| PARK7 |
| PDK1 |
| PDK4 |
| PEX13 |
| PEX3 |
| PFKP |
| PGK2 |
| PHF23 |
| PHYHIP |
| PI4K2A |
| PIK3C3 |
| PIK3CA |
| PIK3CB |
| PIK3R4 |
| PINK1 |
| PLEKHM1 |
| PLOD2 |
| PNPO |
| PPARGC1A |
| PPY |
| PRKAA1 |
| PRKAA2 |
| PRKAB1 |
| PRKAB2 |
| PRKAG1 |
| PRKAG2 |
| PRKAG3 |
| PRKD2 |
| PRKG1 |
| PSEN1 |
| PTPN22 |
| RAB12 |
| RAB1A |
| RAB1B |
| RAB23 |
| RAB24 |
| RAB33B |
| RAB39 |
| RAB7A |
| RB1CC1 |
| RBM18 |
| REEP2 |
| REP15 |
| RFWD3 |
| RGS19 |
| RHEB |
| RIMS3 |
| RNF185 |
| RNF41 |
| RPS27A |
| RPTOR |
| RRAGA |
| RRAGB |
| RRAGC |
| RRAGD |
| S100A8 |
| S100A9 |
| SCN1A |
| SERPINB10 |
| SESN2 |
| SFRP4 |
| SH3GLB1 |
| SIRT2 |
| SLC1A3 |
| SLC1A4 |
| SLC22A3 |
| SLC25A19 |
| SLC35B3 |
| SLC35C1 |
| SLC37A4 |
| SLC6A1 |
| SLCO1A2 |
| SMURF1 |
| SNAP29 |
| SNAPIN |
| SNF8 |
| SNRPB |
| SNRPB2 |
| SNRPD1 |
| SNRPF |
| SNTG1 |
| SNX14 |
| SPATA18 |
| SQSTM1 |
| SRPX |
| STAM |
| STAM2 |
| STAT2 |
| STBD1 |
| STK11 |
| STK32A |
| STOM |
| STX12 |
| STX17 |
| SUPT3H |
| TBC1D17 |
| TBC1D25 |
| TBC1D5 |
| TCIRG1 |
| TEAD4 |
| TECPR1 |
| TECPR2 |
| TFEB |
| TM9SF1 |
| TMBIM6 |
| TMEM203 |
| TMEM208 |
| TMEM39A |
| TMEM39B |
| TMEM59 |
| TMEM74 |
| TMEM93 |
| TNIK |
| TOLLIP |
| TOMM20 |
| TOMM22 |
| TOMM40 |
| TOMM5 |
| TOMM6 |
| TOMM7 |
| TOMM70A |
| TP53INP1 |
| TP53INP2 |
| TRAPPC8 |
| TREM1 |
| TRIM17 |
| TRIM5 |
| TSG101 |
| TXLNA |
| UBA52 |
| UBB |
| UBC |
| UBQLN1 |
| UBQLN2 |
| UBQLN4 |
| ULK1 |
| ULK2 |
| ULK3 |
| USP10 |
| USP13 |
| USP30 |
| UVRAG |
| VAMP7 |
| VAMP8 |
| VDAC1 |
| VMP1 |
| VPS11 |
| VPS16 |
| VPS18 |
| VPS25 |
| VPS28 |
| VPS33A |
| VPS33B |
| VPS36 |
| VPS37A |
| VPS37B |
| VPS37C |
| VPS37D |
| VPS39 |
| VPS41 |
| VPS4A |
| VPS4B |
| VTA1 |
| VTI1A |
| VTI1B |
| WDFY3 |
| WDR45 |
| WDR45L |
| WIPI1 |
| WIPI2 |
| XBP1 |
| YIPF1 |
| ZCCHC17 |
| ZFYVE1 |
| ZKSCAN3 |
| ZNF189 |
| ZNF593 |
| ZNF681 |
| APOL1 |
| ARNT |
| ARSA |
| ATF4 |
| ATF6 |
| ATIC |
| BAG1 |
| BAG3 |
| BAK1 |
| BAX |
| BCL2 |
| BCL2L1 |
| BID |
| BIRC5 |
| BIRC6 |
| BNIP3L |
| C17orf88 |
| CAMKK2 |
| CANX |
| CAPN1 |
| CAPN10 |
| CAPN2 |
| CAPNS1 |
| CASP1 |
| CASP3 |
| CASP4 |
| CASP8 |
| CCL2 |
| CCR2 |
| CD46 |
| CDKN1A |
| CDKN1B |
| CFLAR |
| CTSL1 |
| CX3CL1 |
| CXCR4 |
| DAPK1 |
| DAPK2 |
| DDIT3 |
| DIRAS3 |
| DLC1 |
| DNAJB1 |
| DNAJB9 |
| EDEM1 |
| EEF2 |
| EEF2K |
| EGFR |
| EIF2AK2 |
| EIF2AK3 |
| EIF4EBP1 |
| EIF4G1 |
| ERBB2 |
| ERN1 |
| ERO1L |
| FADD |
| FAS |
| FKBP1A |
| FKBP1B |
| FOS |
| FOXO3 |
| GAA |
| GAPDH |
| GNAI3 |
| GNB2L1 |
| GOPC |
| GRID1 |
| GRID2 |
| HDAC1 |
| HIF1A |
| HSP90AB1 |
| HSPA5 |
| HSPB8 |
| IFNG |
| IKBKB |
| IKBKE |
| IL24 |
| ITGA3 |
| ITGA6 |
| ITGB1 |
| ITPR1 |
| KIAA0652 |
| KIAA0831 |
| KIF5B |
| KLHL24 |
| MAP2K7 |
| MAPK1 |
| MAPK3 |
| MAPK8 |
| MAPK8IP1 |
| MAPK9 |
| MBTPS2 |
| MYC |
| NAF1 |
| NAMPT |
| NCKAP1 |
| NFE2L2 |
| NFKB1 |
| NKX2-3 |
| NLRC4 |
| NRG1 |
| NRG2 |
| NRG3 |
| P4HB |
| PARP1 |
| PEA15 |
| PELP1 |
| PEX14 |
| PPP1R15A |
| PRKAR1A |
| PRKCD |
| PRKCQ |
| PTEN |
| PTK6 |
| RAB11A |
| RAB5A |
| RAC1 |
| RAF1 |
| RB1 |
| RELA |
| RPS6KB1 |
| SAR1A |
| SERPINA1 |
| SIRT1 |
| SPHK1 |
| SPNS1 |
| ST13 |
| TBK1 |
| TMEM49 |
| TNFSF10 |
| TP53 |
| TP63 |
| TP73 |
| TSC1 |
| TSC2 |
| TUSC1 |
| VAMP3 |
| VEGFA |
