# Supplementary material for: Identification of Six Autophagy-Related-lncRNA Prognostic Biomarkers in Uveal Melanoma
Source: Dis Markers. 2021 Aug 12;2021:2401617. doi: 10.1155/2021/2401617 (PMC8379639; doi:10.1155/2021/2401617)
Supplement: Supplementary 2 — Supplementary Table 2: primers for rtPCR. [file 2401617.f2.docx]

Supplementary table 2. Primer sequences in RT-PCR

| lncRNA | Forward | Reverse |
| --- | --- | --- |
| U6 | GCTTCGGCAGCACATATACTAAAAT | CGCTTCACGAATTTGCGTGTCAT3 |
| SOS1-IT1 | GGTTTGTCATCCCAGTCTGC | ATGGCCCAAAGACAACAAAG |
| AC0167747.1 | AAAATTTAGCCGGGCGTAGT | AGCAGGGATTTCCAGGCTAT |
| AC100791.3 | GGCTGGTGGAACAGGTTATG | CACAGGTGTTTACAGGGCATATT |
| AC018904.1 | GCCTACTGGAATGTGGGAAG | GCCAGTTGTGATGGCTCATA |
| AC104825.1 | ATGTGGGACTTCCCTCACTG | AGAACAAACAGGCCACTGCT |
| AC090617.5 | TGCTGTGTCCCTGACCATAA | GCATGGGTGCACATACTCAC |
