# Supplementary material for: Identification of Six Autophagy-Related-lncRNA Prognostic Biomarkers in Uveal Melanoma
Source: Dis Markers. 2021 Aug 12;2021:2401617. doi: 10.1155/2021/2401617 (PMC8379639; doi:10.1155/2021/2401617)
Supplement: Supplementary 3 — Supplementary Figure: coexpression. Coexpression of lncRNA/mRNA shown with a Sankey diagram (A) and Cytoscape (B). [file 2401617.f3.docx]

Supplementary Figure


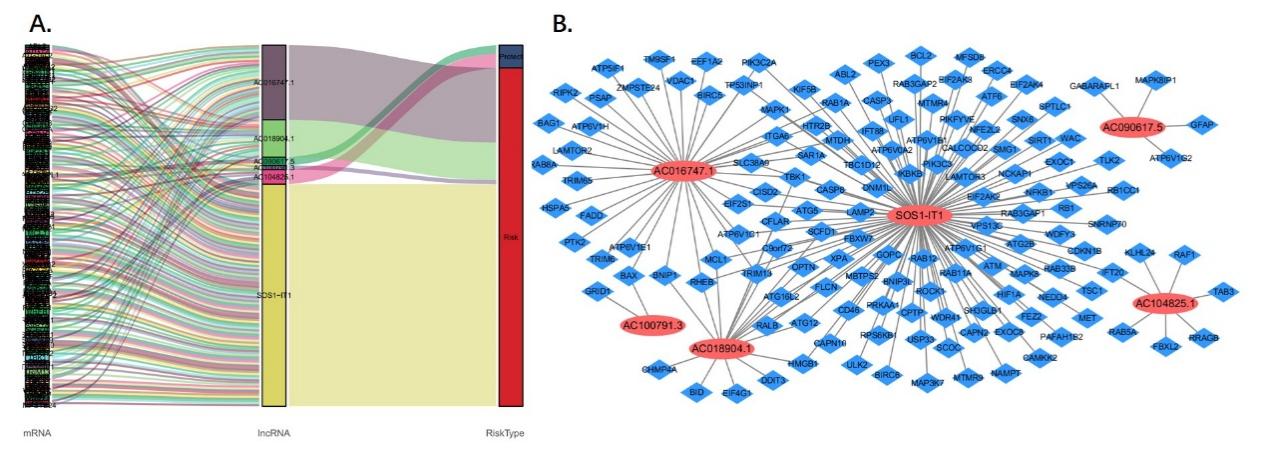


Figure legend: Coexpression. Coexpression of lncRNA/mRNA shown with a Sankey diagram (A) and Cytoscape (B).
